# Retraction: Ikzf2 regulates the development of ICOS+ Th cells to mediate immune response in the spleen of S. *japonicum*-infected C57BL/6 mice

**DOI:** 10.3389/fimmu.2025.1717665

**Published:** 2025-10-08

**Authors:** Frontiers Editorial Office

The journal retracts the August 12^th^ 2021 article cited above.

Following publication, concerns were raised regarding the validity of the data in the article. The authors failed to provide a satisfactory explanation during the investigation, which was conducted in accordance with Frontiers’ policies. Given the concerns, and the lack of raw data, the editors no longer have confidence in the findings presented in the article. The authors do not agree to this retraction. Frontiers would like to thank the users on PubPeer for bringing the published article to our attention.

